# Chikungunya Virus Infection: An Update on Joint Manifestations and Management

**DOI:** 10.5041/RMMJ.10260

**Published:** 2016-10-31

**Authors:** Maria Krutikov, Jessica Manson

**Affiliations:** 1 Department of Infectious Diseases, University College London Hospital, London, UK; 2 Department of Rheumatology, University College London Hospital, London, UK

**Keywords:** Arthritis, arthropod-borne virus, Chikungunya

## Abstract

The advent of sophisticated diagnostics has enabled the discovery of previously unknown arthropod-borne viruses like Chikungunya. This infection has become increasingly prevalent in the last 10 years across the Indian Ocean and has been brought to media attention by a recent outbreak in the Caribbean. The outbreak has been aided by a drastic rise in air travel, allowing infected individuals to transport the virus to previously unaffected regions. In addition, a recently documented viral mutation has allowed its transmission by the *Aedes albopictus* mosquito, therefore facilitating outbreaks in Southern Europe and the USA. The duration and extent of the arthritis seen peri- and post infection has become a topic of academic interest. Although published data are largely observational, there has been a definite increase in original research focusing on this. Symptoms can persist for years, particularly in older patients with pre-existing medical conditions. The etiology is still not fully understood, but viral persistence and immune activation within synovial fluid have been shown in mouse models. There have been no prospective clinical trials of treatment in humans; however, animal trials are in process. The mainstay of treatment remains anti-inflammatories and steroids where necessary. The clinical presentation seems to mimic common rheumatological conditions like rheumatoid arthritis; therefore recent recommendations suggest the use disease-modifying agents as a common practice for the specific syndrome. This review uses recent published data and draws on our own clinical experience to provide an overview of joint complications of Chikungunya infection.

## INTRODUCTION

Chikungunya virus (CHIKV) is a mosquito-borne alpha virus that has precipitated several large outbreaks across the southern hemisphere in the last decade. First described in Tanzania in the 1950s, Chikungunya, also known as break-bone fever, means “that which bends up” in the Makonde language. Following a few outbreaks in the 1960s and 1970s in Asia and Africa, the virus re-emerged in 2005 and spread across the Indian Ocean. Since 2014, over a million cases have been reported in the Americas and the Caribbean, with declining case numbers in 2016. Much of what we currently know about the disease was first published following a large outbreak on the island of La Réunion, affecting 37% of the entire population.^1^

The virus causes a self-limiting acute illness comprising of fever, rash, arthralgia, and myalgia. Other complications, like multi-organ failure and neurological manifestations, have been described but are extremely rare. The most notable long-term complication is a chronic debilitating arthritis that causes considerable disability in certain groups. As we gain more experience with this disease, published literature describing this manifestation has become more plentiful; however, little is understood about the pathophysiology and optimal management of this condition. This review will provide an update of current data and recommendations on Chikungunya-related arthralgia.

## THE VECTOR

Chikungunya is a zoonotic arbovirus that is transmitted by the *Aedes* mosquito. These are day-biting arthropods that breed in open water and can be found across Africa and Asia. Epidemics occur through human–mosquito transmission made possible by high-circulating viremia during acute infection. Between epidemics, the life cycle is maintained through primates and mainly *Aedes aegypti* mosquito. The genetic variation seen in this RNA virus has allowed mutation of the virus to enable transmission by the *Aedes albopictus*, an arthropod that is well established in the Americas and Europe.^2^ This has facilitated the outbreaks of the last 10 years, around the Indian Ocean and across the Caribbean, with an outbreak in Italy in 2007 and cases in other parts of Europe.^3^ Subsequently, there are concerns that the virus may, in time, cause further outbreaks across Europe and Northern America.

## DIAGNOSTICS

Diagnostic techniques have improved significantly in recent years and can be grouped into molecular and serological tests.

Molecular tests are most useful in the acute phase of illness. These use reverse transcriptase PCR assays to amplify fragments of the CHIKV genome and can be used to quantify fragments in real time.^4^ A development in recent years has been the multiplex assay on a chip that can simultaneously detect 26 tropical pathogens including CHIKV and dengue.^5^

Serological assays can be used to determine whether the patient has been previously infected. These use enzyme-linked immunosorbent assays (ELISAs) that capture IgM and IgG. Other tests include immunofluorescence and immunoblot assays for CHIKV proteins.^4^ More recently a rapid immunochromatographic test has been developed that can detect CHIKV, best used in the first 5 days of illness, with a sensitivity of 89.4% and specificity of 94.4%.^6^ This would allow rapid diagnosis at the point of care, therefore precluding the need for further investigations.

## CLINICAL COURSE OF THE DISEASE

The clinical illness has a relatively short incubation period—typically 2–10 days. Sudden-onset high fever is usually the first symptom and can last up to a week and can follow a biphasic course. Fever often precedes a maculo-papular rash over the trunk and extremities, headache, myalgia, and arthralgia. Ocular disease has been described in Malaysia from a few cases of anterior uveitis.^7^ More severe complications include nephritis, myocarditis, meningo-encephalitis, Guillain–Barré syndrome, and cranial nerve palsies that can occur during the acute illness or in subsequent months.^4^ It can be difficult to differentiate acute CHIKV infection from dengue; however, it has been suggested that thrombocytopenia is more indicative of dengue, whereas rash and arthralgia suggest CHIKV.^8^ It has also been noted that pregnant women infected with the virus in the last few days of pregnancy are able to transmit the virus to the baby, which can lead to severe encephalopathy in the neonate and subsequent neurodisability, as demonstrated in a recent cohort study from La Réunion.^9^

After the acute illness, which can last from 7 to 10 days, rheumatic sequelae can persist for months to years. These can have broad clinical presentations and an, as yet, unclear etiology.

## CHIKUNGUNYA RHEUMATISM

Musculoskeletal manifestations of disease have been shown to affect 4%–75% of those infected with CHIKV.^4^ These figures vary widely depending on baseline genetic susceptibility of populations, cultural perceptions, and quality of study.

Arthralgia usually affects more than one joint, particularly knees, ankles, hands, and wrists in a bilateral and symmetric distribution.^10^ In some cohorts, over 50% of patients had arthralgia and clinically detectable joint swelling at 3 years after their acute infection.^11^,^12^ A 6-year retrospective study in La Réunion by Javelle et al. looked at patients referred to a rheumatologist due to rheumatic symptoms lasting more than 4 months following CHIKV infection. Out of 159 cases, they found that 59% met the criteria for *de novo* chronic inflammatory rheumatism (CIR) like rheumatoid arthritis, spondylarthropathy, and undifferentiated polyarthritis, and 31% had pre-existing rheumatic musculoskeletal disorders. Amongst those with *de novo* rheumatoid arthritis, 80% developed joint damage within 3–4 years. They found that some patients remained symptomatic for 6–8 years. Patients that did not fit the CIR criteria presented most commonly with bilateral distal polyarthralgia, fibromyalgia, edema, and carpal tunnel syndrome.^13^ This finding was mirrored in a study from Sri Lanka from 2006 where 21% of the total number of infected individuals exhibited carpal tunnel syndrome.^14^ There have also been recent cases of patients presenting with catastrophic antiphospholipid syndrome and adult-onset Still’s disease.^15^

Plasma markers of inflammation such as ESR and CRP are unreliable in predicting the severity of joint involvement. Javelle et al. found that only 2 out of 15 patients with spondylarthropathies were HLA-B27 positive, both of whom had psoriasis. Rheumatoid factor (RF) or anti-citrullinated protein anti-body (ACPA) positivity was found in 30% of patients, which was similar to other studies.^13^

Radiological imaging of affected joints showed erosions and joint space narrowing in a small cohort in La Réunion.^16^ Destructive lesions were more commonly seen in those with an rheumatoid arthritis-like presentation, with up to 80% affected in some cohorts.^13^ By contrast, in our London cohort of CHIKV-infected returning travelers, we found predominantly joint effusions rather than synovitis on ultrasound examination (unpublished data). Magnetic resonance imaging has similar findings to ultrasound with predominant joint effusion, synovial thickening, tendonitis, and tenosynovitis.^17^

Risk factors for prolonged disease are age greater than 50 years, longer acute stage of illness (more than 15 days), and presence of other comorbidities.^18^ A study looking at predictors of rheumatism in the TELECHIK cohort of patients in La Réunion performed a multinomial logistic regression on the 346 patients with declared rheumatic musculoskeletal pain. They found age greater than 45, severe rheumatic involvement at presentation (fever, more than six joints affected, and four other rheumatic symptoms), and CHIKV-specific IgG titers were strong determinants of persistent musculoskeletal pain. The CHIKV-specific IgG titers were correlated to age, female gender, and severity of initial rheumatic symptoms. They concluded that the humoral immune response has a significant prognostic value; however, the adaptive immune response plays an important role in chronic manifestations.^10^

The etiology of the arthralgia is not fully understood. Research from mouse models comparing wild-type and *Rag1-*deficient mice that lack T and B cells inoculated with CHIKV shows higher viral levels in *Rag1-*deficient mice. This suggests the role of the adaptive immune system in host response and persistence of infection. In addition, tissues with high CHIKV RNA levels in these mice histologically exhibited synovitis, arthritis, and tendonitis, suggesting that the arthritis is not mediated by the adaptive immune system. The persistence of CHIKV was tissue-specific as sampling of other organs showed rapid clearance of the virus when compared with joints and skeletal muscle, in which it persisted for at least 16 weeks.^19^

Samples from a human synovial biopsy taken from an affected individual with chronic joint disease showed joint infiltration with natural killer cells, CD4 cells, and CHIKV RNA in macrophages. Histologically there was synovial hypertrophy, vascular proliferation, and perivascular macrophage infiltration similar to that seen in rheumatoid arthritis.^20^ This suggests that the CHIKV arthralgia is etiologically similar to other inflammatory arthropathies and, as such, a similar treatment approach can be adopted.

As disease can last for years, there is evidence that quality of life of individuals is adversely affected. A study by Couturier et al. followed up CHIKV-infected individuals for 2 years following diagnosis. Of the 391 patients that answered the survey, 55% of patients considered themselves to have not recovered from CHIKV at a median of 23.4 months post diagnosis. Assessments of quality of life using well-known tools like SF-36 (short term health survey), GHQ-12 (general health questionnaire), and AIMS2-SF (arthritis impact measurement scale) were performed at regular intervals and compared with age- and gender-matched scores. Those who considered themselves as unrecovered had much lower scores than recovered participants.^18^ In addition, there is evidence of the financial burden that this can have—a study from La Réunion has estimated the annual cost at €34 million.^12^

## TREATMENT AND PREVENTION OF INFECTION

There are currently no licensed treatments for CHIKV infection. Several drugs have been found to have modest effect by targeting viral replication or host cellular components. Although these mainly target acute viral infection, little is known of the effect of antiviral treatments on chronic infection.

Drugs that inhibit viral entry include chloroquine and arbidol. Chloroquine, historically an anti-malarial drug, has been thought to be effective against chronic CHIKV since the early 1980s; however, a recent trial using it as treatment in acute infection has shown it to be ineffective.^21^,^22^ Arbidol is a licensed antiviral in Russia, and arbidol analogues have been found to have anti-CHIKV activity.^23^

A potent inhibitor of viral protein translation has been found to be harringtonine and its more stable analogue, homoharringtonine. This drug has minimal cytotoxic effects and has recently been licensed in the USA for treatment of acute myeloid leukemia.^24^

Most effective of the antiviral drugs are those that target viral genome replication. Ribavirin has been used most widely as it is licensed for treatment of respiratory syncytial virus in infants. Ribavirin in combination with interferon has been shown to act synergistically in preventing CHIKV replication.^25^

Mycophenolic acid appears to be more effective than ribavirin *in vitro* as described by Khan et al. in 2011.^26^ Another anti-metabolite that is effective *in vitro* is 6-azauridine as it inhibits synthesis of pyrimidines.^22^,^25^ Favipiravir has been shown to protect mice against CHIKV-caused disease as a potent polymerase inhibitor.^27^

Although these drugs have displayed some efficacy, they can only reduce viral replication by 50% in mouse models and have not yet moved to more relevant testing platforms for chronic CHIKV infection.

An alternative to these antiviral drugs is human poly- or monoclonal antibodies. The use of human polyvalent antibodies taken from human plasma donors in the convalescent phase of CHIKV infection showed full protective efficacy in mouse models. This effect was completely therapeutic 8 hours post infection and reduced as time passed.^27^ Prophylactic administration of neutralizing monoclonal antibodies to *Rag1*-deficient mice prevented them from developing persistent infection and was even effective in clearing CHIKV from tissues when administered later on in infection.^19^ This work suggests the potential for monoclonal neutralizing antibodies in prevention of CHIKV-associated arthralgia in endemic areas.

Further preventive measures such as vaccines against CHIKV have not yet been licensed but have been a goal since the 1960s. The CHIK-IRES vaccine is an attenuated vaccine derived from a strain of virus from La Réunion in 2006. This has been engineered to prevent it from replicating in the *Aedes* mosquito and, following recent trials in non-human primates, is planned for phase I trials. Another promising vaccine has implemented induced neutralizing antibodies and virus-like particles (VLPs) which are immunogenic in non-human primates and has entered into phase II trials (NCT02562482).^27^ It will be important to show that these vaccines can have long-lasting protection against the virus and are robust against wild-type virus and different vectors.

The release of a genetically engineered mosquito carrying lethal genes (Release of Insects with Dominant Lethal Gene [RIDL]) has also been investigated for prevention of infection. However, there have been concerns that this measure in *Aedes aegypti* in Panama has allowed the mutation of CHIKV to be transmitted by the *Aedes albopictus*.^28^ In addition, it may even drive selection of more virulent and severe viruses.^2^ Another approach has been to infect mosquitoes with the *Wolbachia* bacterium, which is thought to interfere with mosquito replication.

## MANAGEMENT OF CHIKV RHEUMATISM

Management of CHIKV rheumatic disease has traditionally used a generic approach to this diverse group of clinical presentations. Javelle et al. recently suggested grouping patients according to their clinical syndrome and tailoring treatment accordingly.^13^

The mainstay of treatment has been with anti-inflammatory drugs, physiotherapy, and short courses of oral steroids; however, clinical withdrawal of these treatments can be difficult. Evidence from La Réunion suggested that loco-regional disease responded well to physiotherapy and to localized joint injections. Carpal tunnel syndrome was treated with NSAIDs and with physiotherapy. Urate-lowering drugs can be effective, as can bone-strengthening measures such as vitamin D.^13^

In those with persistent symptoms, there is little evidence on effective therapies. Several disease-modifying drugs (DMARDs) have been trialed with varying success. Chloroquine has some antiviral effect but has not been found to be more effective than other anti-inflammatories like meloxicam in acute and chronic CHIKV arthralgia.^29^ Methotrexate has been widely used, particularly in patients who present with a systemic polyarthritis. A recent study showed that 75% of patients had a positive clinical response to this.^13^ Sulfasalazine has been shown to have good clinical efficacy, particularly when combined with methotrexate.^30^

There are no good-quality trials assessing the use of biologic drugs in CHIKV rheumatic disease. There are data to suggest that serum cytokines are raised in patients with persistent symptoms, including IL-6 and IL-8.^31^ In addition, a number of cases report successful use of biologic immunomodulatory agents such as infliximab or etanercept in patients with severe disease.^32^

Bindarit is a small-molecule indazolic derivative that has anti-inflammatory properties through inhibiting chemokine synthesis. This has been trialed in CHIKV mouse studies to reduce joint damage and inflammation and improve bone health.^33^,^34^ These studies showed reduced macrophage infiltration of joints and therefore reduced tissue damage and disease symptoms and also reduced osteoclastic activity. As it is less immunosuppressive than more widely used biologic agents such as anti-TNF drugs, it has the potential to be a safe treatment to prevent viral-induced joint damage and bone loss.

## CONCLUSION

Since its initial description in the 1950s, there have been multiple large Chikungunya outbreaks worldwide. Mutation of the virus to allow carriage by *Aedes albopictus* has enabled transmission of the virus in previously unexposed areas, substantially increasing the at-risk population. The recent warnings in relation to Zika virus have emphasized the increasing risk from *Aedes*-borne viruses to the general population.

The growing body of evidence regarding the debilitating chronic arthritis following Chikungunya infection has illustrated trends in presentation. It has also allowed certain conclusions to be drawn regarding treatment strategies from the experience of clinicians worldwide. As the arthritis often has a strong semblance to well-described rheumatic presentations, it has often been treated as such, with reasonable outcomes. The importance of maintaining a holistic approach and considering quality of life is evident, particularly as long-term sequelae are more frequently seen in older patients with pre-existing medical comorbidities. Although the mortality associated with this infection is small, the morbidity and burden of disease is large, affecting several million people worldwide. With a rapidly expanding area of endemicity, rheumatologists should be prepared to see more chronic arboviral arthritides in their day-to-day clinics. More research and awareness is necessary in order to develop better treatment strategies for such patients.

## Abbreviations

CHIKV: Ckikungunya virus
CIR: chronic inflammatory rheumatism
